# Palm Weevil Larvae (*Rhynchophorus phoenicis* Fabricius) and Orange-Fleshed Sweet Potato-Enriched Biscuits Improved Nutritional Status in Female Wistar Albino Rats

**DOI:** 10.1155/2020/8061365

**Published:** 2020-04-08

**Authors:** Jessica Ayensu, Christopher Larbie, Reginald Adjetey Annan, Herman Lutterodt, Anthony Edusei, Su Peng Loh, Ernest Amponsah Asiamah

**Affiliations:** ^1^Department of Biochemistry and Biotechnology, Kwame Nkrumah University of Science and Technology, Kumasi, Ghana; ^2^Department of Clinical Nutrition and Dietetics, University of Cape Coast, Cape Coast, Ghana; ^3^Department of Food Science and Technology, Kwame Nkrumah University of Science and Technology, Kumasi, Ghana; ^4^School of Public Health, Kwame Nkrumah University of Science and Technology, Kumasi, Ghana; ^5^Department of Nutrition and Dietetics, Universiti Putra, Seri Kembangan, Selangor, Malaysia; ^6^Department of Forensic Science, University of Cape Coast, Cape Coast, Ghana

## Abstract

Edible insects have emerged as an inexpensive alternative source of protein for reducing the burden of malnutrition worldwide. However, there is a dearth of evidence on its efficacy, and thus, the aim of this study was to investigate the effect of edible insect consumption on the nutritional status of female Wistar albino rats. The study assessed the subchronic effect of palm weevil larvae (PWL) and orange-fleshed sweet potato- (OFSP-) enriched biscuits (fortified biscuits (FB), plain biscuits (PB), biscuits fortified with PWL (PWB), and biscuits fortified with OFSP only (SPB)) as a model to predict the potential of PWL to improve the nutritional status of pregnant women in Ghana. Twenty-five female Wistar albino rats were randomly assigned to five experimental groups to receive one of the five feed supplements for 28 days. After which, the effects of treatment on haematological and biochemical parameters including lipid profile were assessed. No significant differences were observed with haematological (Hb) parameters. However, total cholesterol levels of the FB, PB, PWB, and SPB were significantly higher than in the N group. Apart from elevated total cholesterol concentrations, biscuits fortified with PWL had no adverse effects and can be a nutritious snack for maintaining acceptable HB levels.

## 1. Introduction

Adequate nutrition prior to and during pregnancy is a cornerstone for desirable maternal and child outcomes. Globally, maternal malnutrition is a challenge with important repercussions for the incidence of acute and chronic diseases and the economic efficiency of individuals and societies. In low- and middle-income countries, deficiencies of essential nutrients such as iron, protein, zinc, folate, calcium, and vitamin A are highly prevalent among pregnant women. These deficiencies result in anaemia, impaired immune functions, low birth weight, intrauterine growth restrictions, decreased motor activity, and impaired cognitive [1–4]. In all cases, significant associations have been drawn between food insecurity, poor nutrient absorption, and micronutrient deficiencies [5, 6]. To combat this problem, food-based interventions along with already existing supplementation and fortification programmes are appropriate and sustainable approaches for curbing maternal malnutrition and its consequences.

Animal protein foods are better sources of bioavailable micronutrients like zinc and iron, and for many years, livestock production has been a major source for meeting human protein and micronutrient requirements. However, due to an estimated population increase, cost of conventional protein production, and scarcity of land, the Food and Agricultural Organisation has predicted a future deficit in animal protein supply [7]. In developing countries where dietary protein sources are largely limited to plant foods due to the high cost of conventional protein [7, 8], this foreseen deficit in animal protein supply may increase malnutrition incidence.

As part of the many strategies and suggestions outlined to overcome this foreseen scarcity, the FAO has encouraged entomophagy [9]. Edible insects have high nutritive value and constitute a promising healthy source of food high in fat, protein, vitamins, minerals, and fibre [9–11]. According to the FAO, the omega 3 and 6 content of mealworms is comparable to that in fish and higher than that in pigs and cattle and its protein, vitamin, and mineral contents are comparable to fish and meat [9].

In Ghana, where malnutrition is a pressing issue particularly for women and children, the larvae of the African palm weevil (*Rhynchophorus phoenicis* Fabricius 1801), locally known as “akokono,” a component of traditional cuisines, is being promoted for preventing protein-energy malnutrition and possibly anaemia in women and children. Palm weevil larvae (PWL) is an excellent source of amino acids, fatty acids, vitamin B12, and minerals such as zinc, iron, potassium, and phosphorus [12, 13].

Orange-fleshed sweet potato is a well-known food with high potential for alleviating vitamin A deficiency (VAD) in sub-Saharan Africa. It is known to have an excellent amount of highly bioavailable beta-carotene. Since its introduction in the subregion, many published articles and reports have revealed its efficacy for combating VAD in many African countries [14]. In Uganda, Kenya, Ethiopia, and Mozambique, orange-fleshed sweet potato focused studies revealed improved vitamin A intake and status among children and mothers as well as reduced child diarrhoea [14–18]. However, it is underutilized in Ghanaian cuisines due to low publicity [19, 20].

Although the consumption of edible insects is gaining grounds in several parts of the world with many research evidences on their rich nutrient composition backing this practice, concerns have been raised about the bioavailability of the nutrients for metabolic processes as well as possible adverse effects associated with the practice. In the case of Ghana where the inclusion of palm weevil larvae in meals is being promoted among the vulnerable as an alternative animal source food for preventing anaemia, there is paucity of data on its efficacy. Moreover, considering the obvious and common knowledge on the relation between dietary saturated fat consumption and cardiovascular disease risk and the fact that palm weevil larvae are composed of 65% fat (dry weight), the objective of this study was to assess the effects of edible insect consumption on the nutritional status of female rats to inform its use among pregnant women in Ghana in future studies. The study was designed to investigate the effects of biscuits fortified with PWL and orange-fleshed sweet potato on the nutritional status as well as possible adverse effects associated with its consumption in female rats.

## 2. Materials and Methods

### 2.1. Intervention Feed

Four biscuits with varying caloric contents were produced from wheat flour, palm weevil larvae flour (PWLF), and orange-fleshed sweet potato (OFSP). Details of the recipe, nutrient composition, safety, and acceptability are discussed elsewhere [21]. In brief, farmed palm weevil larvae were obtained from the ASPIRE Food Group breeding site, Kwame Nkrumah University of Science and Technology, Kumasi, Ghana. The larvae were washed, parboiled, and oven-dried at 60°C to constant weight. The dried larvae were then milled to obtain a powdery consistency. The orange-fleshed sweet potato flour and other ingredients were purchased from a local market in Kumasi, Ghana. The palm weevil larvae flour (PWLF) and the orange-fleshed sweet potato flour (OFSPF) were mixed with wheat flour in four different formulations to obtain four composite flours which were then mixed with other ingredients (sugar, shortening, bicarbonate of soda, salt, and flavoring) to obtain a dough. The dough was then placed on a cutting board and rolled out until uniform thickness and textures were obtained. Biscuit cutter was used to cut the sheet of rolled dough into desired shapes and sizes and then baked in an oven at 220°C for about 15 min. The cooked biscuit was allowed to cool at room temperature, packed, and stored according to Okaka [22]. Nutrient composition of the four intervention biscuits and the standard rat chow is shown in Table 1.

Wistar albino female rats weighing between 75 g and 120 g, obtained from the animal facility of the School of Veterinary Medicine, University of Ghana, Legon, Ghana, were used for the study.

### 2.2. Experimental Design

**Figure 1:**
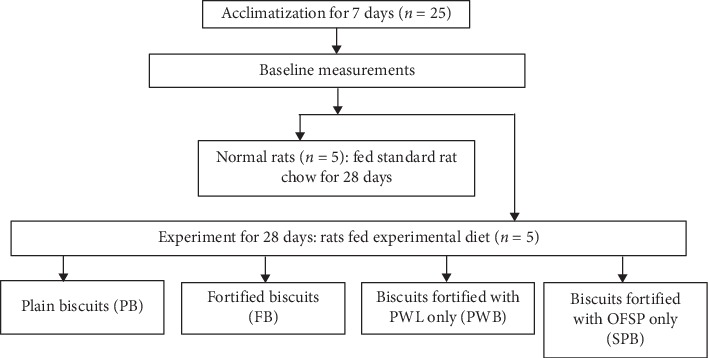
Experimental groups.

In this study, 25 animals were randomly assigned to 5 experimental groups to receive at least 50 g of one of the four intervention biscuits for 28 days as shown in Figure 1. The animals were group-housed in cages bedded with wood shavings and kept at the Animal Facility of the Department of Biochemistry and Biotechnology, Kwame Nkrumah University of Science and Technology, Kumasi, Ghana, throughout the study. They were kept under standard laboratory conditions of temperature and humidity with a daily 12 h light : dark cycle throughout the study. The rats were also provided with standard rat chow and distilled water ad libitum during a one-week period (7 days) of acclimatization before the commencement of the experiment. The animal experiments were conducted according to guidelines of the Committee for the Purpose of Control and Supervision of Experiment on Animals (CPCSEA, New Delhi, India) and the Guide for Care and Use of Laboratory Animals (NRC, 2011). All animals were humanely handled during the experiment.

### 2.3. Effect of Treatment on Body Weight Changes

Animals in each group were weighed on the first day (D0) and thereafter, at the end of every four days (D4, D8,…, D28) using a weighing scale (Ohaus CR221 Portable Scale). The percent change in body weight was calculated using the following formula:

(1) $$\begin{array}{c} \text{Percent change in body weight}=\;\frac{{\text{weight}}_{n}-{\text{weight}}_{o}}{{\text{weight}}_{o}}\times 100, \end{array}$$

where weight_*n*_ is the weight on Day 4, D8,…, D28 and weight_*o*_ is the weight on the first day (D0).

### 2.4. Effect of Treatment on Haematological and Biochemical Parameters

After 28 days of treatment, the animals were euthanized by ether anesthesia after an overnight fast. Incisions were quickly made in the cervical regions of euthanized animals using sterile blade and blood collected into EDTA tubes for haematology analysis using Sysmex Haematology System (USA). Parameters analysed included haemoglobin (HB), haematocrit (HCT), white blood cell (WBC) count, red blood cell (RBC) count, mean corpuscular volume (MCV), mean corpuscular haemoglobin (MCH), mean corpuscular haemoglobin concentration (MCHC), and platelet (PLT). Part of blood samples were collected into gel-activated tubes, left to clot, and centrifuged for 10 minutes at 3500 rpm. The serum obtained was analysed for alanine aminotransferase (ALT), total protein, albumin, creatinine, urea, triglycerides, total cholesterol, and high-density lipoproteins (HDL) cholesterol using the Selectra E (Vital Scientific, Japan) and reagents from ELITECH (France).

### 2.5. Effect of Treatment on Organ Weight and Uterus Histology

The liver, kidney, and uterus of rats were washed in buffered normal saline and weighed to obtain the absolute liver, kidney, and uterus weights. Relative weights were calculated using the formula:

(2) $$\begin{array}{c} \text{Relative organ weight }=\frac{\text{absolute organ weight}}{\text{body weight at sacrifice }}\times 100. \end{array}$$

Uteri were preserved in formalin and processed with an automated tissue processor (Leica TP 1020, Germany) after which they were embedded in paraffin wax. The tissue blocks were slit into serial sections using the rotary microtome, deparaffinized, and later stained with periodic acid-Schiff (PAS) stain (Bancroft and Gamble, 2008). The microscopic architecture of epithelial cells, stratum compactum, and the endometrial glands of the endometrium of the uteri of experimental rats on the PAS-stained slides were histopathologically examined by a pathologist.

### 2.6. Statistical Analysis

Data were analysed using GraphPad Prism 8 for Windows. The experimental results were expressed as the mean ± standard error mean (SEM). Differences between groups were determined using ANOVA followed by Tukey's test. Values for which *p* < 0.05 were considered as statistically significant.

## 3. Results

### 3.1. Food Intake and Body Weight Gain

**Figure 2:**
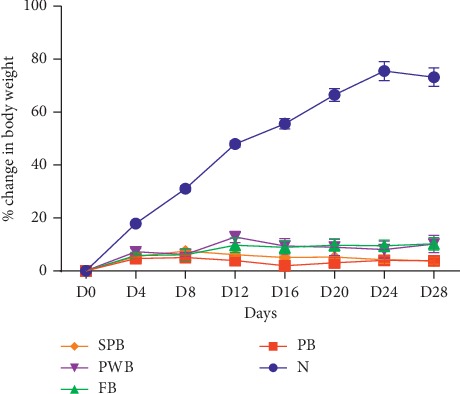
Effect of treatment on body weight. Data are presented as means ± SEM, *n* = 5. N = normal/standard rat chow, PB = plain biscuits, FB = biscuits fortified with PWL and OFSP, PWB = biscuits fortified with PWL only, and SPB = biscuits fortified with OFSP only.

The effect of the intervention on the body weight is shown in Figure 2. The weight change in the PB, FB, PWB, and SPB groups was not significantly different. However, the weight change in the N group was significantly higher than the change in the FB, PWB, PB, and SPB groups.

### 3.2. Effect of Treatment on Relative Organ Weight

**Figure 3:**
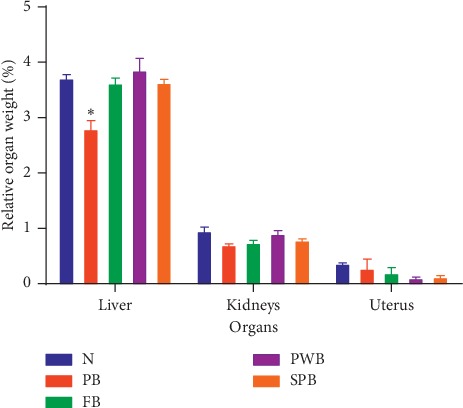
Effect of treatment on relative organ weight of animals in the control and treated groups. Data are presented as means ± SEM, *n* = 5, ^*∗*^significant at *p* < 0.001 according to ANOVA. N = normal/standard rat chow, PB = plain biscuits, FB = biscuits fortified with PWL and OFSP, PWB = biscuits fortified with PWL only, and SPB = biscuits fortified with OFSP only.

The effect of the intervention on the relative organ weight of animals is presented in Figure 3. No significant changes in the weight of the kidneys and uterus in all groups were observed. However, the mean liver weight for the FB, PWB, and SPB groups was significantly higher than the weight for the PB group (*p* < 0.001).

### 3.3. Effect of Treatment on Haematological Parameters

The mean haemoglobin levels for the PB group (11.96 ± 0.42 g/dl) were significantly lower than the mean levels for the FB (13.18 ± 0.28 g/dl), PWB (12.88 ± 0.33 g/dl), and SPB (13.24 ± 0.04 g/dl) groups. However, the mean haemoglobin levels in the FB, PWB, and SP groups were not significantly different from the concentrations in the N group. The mean haematocrit concentration in the PB and PWB groups was significantly different from the FB and SPB groups. Among all the supplemented groups, the FB group had the highest haematocrit concentration although this was not significantly different from the SPB. For RBC, MCHC, and PLT, no significant difference was observed between the groups (Table 2).

### 3.4. Effect of Treatment on Biochemical Parameters of Animals

The effects of the nutritional interventions on the lipid profile of animals are shown in Table 3. The mean serum total cholesterol (TG) levels for the supplemented groups ranged from 2.26 ± 0.13 mmol/L in the PB group to 3.04 ± 0.3 mmol/L in the PWB group. The mean total cholesterol levels in the PB, FB, PWB, and SPB groups were not significantly different. Although the mean total cholesterol levels in the supplemented groups were not significantly different, they were significantly higher than that of the N group. The mean serum low-density lipoprotein (LDL) levels ranged from 1.36 ± 0.13 mmol/L in the PB group to 1.94 ± 0.35 mmol/L in the PWB group. Although the mean LDL concentration in the PWB group was higher, it was not significantly different from the levels in the PB, FB, and SPB groups.

Table 4 shows the effects of treatment on some biochemical indicators of animals in the control and treated groups. The serum urea levels ranged from 6.42 ± 0.62 mmol/L in the PB group to 7.62 ± 0.25 mmol/L in the PWB group. Although the urea levels in the PB group were the lowest, it was not significantly different from the levels in the FB, PWB, and SPB groups. As regards creatinine levels, no significant difference was observed in all the intervention groups (PB, FB, PWB, and SPB). The mean ALT concentrations in the PB group (84.0 ± 12.0 *μ*/L) were significantly higher than the concentrations in the FB, PWB, and SPB groups. Total protein and globulin levels were significantly lower in the PB group compared to the levels in the FB, PWB, and SPB groups.

### 3.5. Histopathological Evaluation

**Figure 4:**
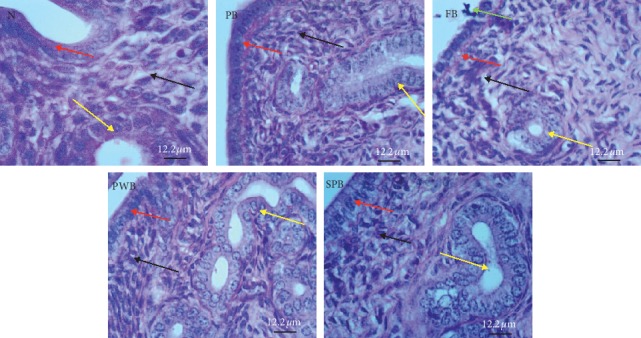
Light micrographs of endometrium from PAS-stained uterine sections. N (control/standard rat feed); PB (plain biscuits); FB (biscuits fortified with OFSP and PWL); PWB (biscuits fortified with palm weevil only), and SPB (biscuits fortified with OFSP only). Red arrow represents endometrial epithelial cells, black arrow represents stratum compactum, yellow arrow represents endometrial glands, and green arrow represents pyknotic nuclei.

Histopathological examination of the uterus from the PB group showed low columnar epithelial cells with traces of basal vacuolations and straight endometrial glands. Low epithelial cells with no distinct cilia and microvilli but basal vacuolations, collagenous stratum compactum with traces of vasodilatation, and disintegrated endometrial glands and pyknotic nucleus were observed in the FB group. The PWB and SPB groups had slightly tall epithelium, tortuous endometrial gland with slight secretion, and its cells having nucleus with prominent nucleolus and tall endometrial epithelial cells with vacuolated cytoplasm, less compact stratum compactum, and tortuous endometrial gland with secretion, respectively. These observations are not significantly different from expected observations in the N group (Figure 4).

## 4. Discussion

Humans have consumed insects and insect products like honey for thousands of years. Edible insects have received a lot of recognition from regional and subregional organisations for their rich protein, essential fatty acids, fibre, minerals, and vitamin contents [23] as well as their potential of being a promising avenue through which malnutrition and food insecurity can be sustainably addressed globally [10]. Apart from the many potential benefits of consuming edible insects, several concerns regarding safety, effects of processing, acceptability, and nutrient bioavailability have been raised. Orange-fleshed sweet potato is an inexpensive source of beta-carotene for preventing vitamin A deficiency. In recent times, edible insects have been merged with popular foods to make them more acceptable. We therefore examined the effects of biscuits fortified with palm weevil larvae and orange-fleshed sweet potato on the nutritional status of female Wistar albino rats. This included the effect on body weight, haematology, biochemical parameters, gross anatomical observations on some organs, and histological observations on the uterus.

Generally, the fortified biscuits were well accepted by the animals with an average consumption of 6.4 g, 8.1 g, 5.6 g, and 7 g per day per each animal in the PB, FB, PWB, and SPB groups, respectively, compared to 15.5 g in the N group. Although no episodes of diarrhoea, negative dermatological manifestations, and even death were recorded, the treatment resulted in decreases in body weight of animals receiving the experimental biscuits. Thus, though treatments were well accepted, the food efficiency was reduced in treated groups compared to the normal group (Figure 3). The weight loss in the experimental groups could have resulted from inadequate food intake [24].

Although haemoglobin concentrations in the FB, PWB, and SPB groups were comparable to the N group, the use of more sensitive indicators of haematological status like ferritin and serum iron would have provided a more concrete result since haemoglobin levels in female rats are often affected by hormonal changes. Nevertheless, this result is consistent with that of [20]. This also means that the protein and iron levels of the formulated biscuits are adequate for maintaining acceptable haemoglobin levels and may be effective for anaemia prevention.

A significant increase in the total cholesterol concentrations in the PB, FB, PWB, and SPB groups compared to the N group was observed. The high total cholesterol levels observed can be attributed to the fact that fat is a major ingredient in biscuits and foods that are high in saturated fats have the potential of increasing serum total cholesterol levels [25]. Additionally, palm weevil larvae are composed of 65% fat, majority of which are saturated [26] and this can explain the high total cholesterol levels in the FB and PWB groups. Because the intervention biscuits contained other fat sources, we cannot explicitly state that the increased total cholesterol levels in the FB and PWB groups were induced by the palm weevil larvae. We are also unable to confirm the assertion by Payne et al. [27] that palm weevil larva is a potential culprit for noncommunicable diseases related to overnutrition. Findings of this study showed no adverse effect of palm weevil larvae on the kidneys since the urea and creatinine levels in the PB, FB, PWB, and SPB groups were not significantly different from the N group. Again, the ALT levels in all the intervention groups except the PB group were not significantly different from the N group. Elevated ALTs are often associated with nonalcoholic liver diseases with risk factors being obesity, high serum cholesterol levels, and type II diabetes. The cause of the elevated ALT in the PB group is unclear, but the results of the study suggest fat accumulation in the liver of the PB group [28].

Uteri of all treated animal groups showed the normal histological structure of the endometrium of uterine sections of all the groups. This result suggests no inflammations nor adverse impact on the uterus of the experimental rats.

Presently, only a handful of human studies have assessed the impact of edible insect consumption on nutritional status and most of their results highlight the malnutrition preventing potential of edible insects [29–31]. In a study which assessed the impact of complementary foods fortified with termites on iron levels and anaemia in Kenyan children, Konyole [29] recorded fewer cases of anaemia in the intervention group (26%) than in the control group (50%, *p*=0.006). Although the results of animal studies may not always be suitable for human translation, the findings of this study support that palm weevil larvae have potential for preventing anaemia and thus, that biscuits enriched with palm weevil larvae can be a useful snack for improving protein intake in humans. Our findings contribute to current efforts to provide evidence in support of edible insects as alternative source of protein for combating malnutrition among vulnerable populations. Furthermore, this is the first study to evaluate the impact of palm weevil larvae consumption on the lipid profile and kidney and liver function, although entomophagy has been in existence for many years.

## 5. Conclusion

We conclude that biscuits fortified with OFSP and PWL can be an alternative feed for preventing anaemia and that apart from the significant increase in total cholesterol which may be needful in situations where individuals consistently have very low cholesterol levels and need to increase it within healthy ranges to promote metabolism of hormones, bile acids, and vitamin D, and human plasma cholesterol levels, no other adverse effects were found. Compared to plain biscuits, biscuits enriched with palm weevil larvae and orange-fleshed sweet potato can be considered as a ready source of energy, protein, and micronutrients in emergency situations.

## Figures and Tables

**Table 1:** Nutrient composition of intervention feeds.

Nutrient	N	PB	FB	PWB	SPB
Protein (%)	17	8.01	9.63	10.8	7.16
Fat (%)	3.65	23	34	38.43	21.41
Fibre (%)	3.6	3	4	1.78	2.43
Calcium (mg)	3.5	29.81	31.56	33.5	28.5

N = normal/standard rat chow, PB = plain biscuits, FB = biscuits fortified with PWL and OFSP, PWB = biscuits fortified with PWL only, and SPB = biscuits fortified with OFSP only.

**Table 2:** Effect of treatment on haematological parameters of animals in the control and treated groups.

Parameters	N	PB	FB	PWB	SPB
HB (g/dL)	13.95 ± 0.36^b^	11.96 ± 0.42^a^	13.18 ± 0.28^b^	12.88 ± 0.33^b^	13.24 ± 0.04^b^
WBC (×10^3^/*μ*L)	9.95 ± 1.10^a^	9.88 ± 0.66^a^	11.58 ± 0.86^a^	11.26 ± 2.08^a^	9.30 ± 0.93^a^
RBC (×10^6^/*μ*L)	7.45 ± 0.12^a^	7.14 ± 0.19^a^	7.85 ± .17^a^	7.41 ± 0.19^a^	7.84 ± 0.84^a^
HCT%	50.60 ± 1.11^b^	43.7 ± 1.21^a^	47.53 ± 0.94^a^	45.24 ± 1.30^a^	47.16 ± 0.24^b^
MCV (fl)	67.88 ± 1.43^b^	61.2 ± 0.32^a^	60.58 ± 0.37^a^	61.0 ± 0.65^a^	60.28 ± 0.62^a^
MCH (pg)	18.75 ± 0.24^b^	16.76 ± 0.47^a^	16.78 ± 0.09^a^	17.40 ± 0.34^a^	16.92 ± 0.15^a^
MCHC (g/dL)	27.60 ± 0.27^a^	27.38 ± 0.68^a^	27.74 ± 0.04^a^	28.50 ± 0.41^a^	28.06 ± 0.06^a^
PLT (×10^3^/*μ*L)	773.8 ± 273.44^a^	819.0 ± 123.21^a^	892.8 ± 4.30^a^	904.2 ± 78.76^a^	1032 ± 155.16^a^

HB: haemoglobin; HCT: haematocrit; WBC: white blood cell count; RBC: red blood cell count; MCV: mean corpuscular volume; MCH: mean corpuscular haemoglobin; MCHC: mean corpuscular haemoglobin concentration; PLT: platelet. Means with different letters in a row differ, *p* < 0.05. N = normal/standard rat chow, PB = plain biscuits, FB = biscuits fortified with PWL and OFSP, PWB = biscuits fortified with PWL only, and SPB = biscuits fortified with OFSP only.

**Table 3:** Effect of treatment on lipid profile of animals in the control and treated groups.

Groups	N	PB	FB	PWB	SPB
Tol cholesterol (mmol/)	1.75 ± 0.13^b^	2.26 ± 0.13^a^	2.72 ± 0.17^a^	3.04 ± 0.30^a^	2.82 ± 0.15^a^
LDL (mmol/L)	0.96 ± 0.25^a^	1.36 ± 0.13^a^	1.81 ± 0.21^a^	1.94 ± 0.35^a^	1.84 ± 0.22^a^
HDL (mmol/L)	0.68 ± 0.11^a^	0.54 ± 0.07^a^	0.58 ± 0.07^a^	0.74 ± .06^a^	0.72 ± 0.08^a^
Triglycerides (mmol/L)	0.78 ± 0.06^a^	0.74 ± 0.05^a^	0.72 ± 0.09^a^	0.80 ± 0.12^a^	0.58 ± 0.91^a^

Data presented are means and standard errors of lipid profiles of animal groups. Tol cholesterol: total cholesterol; LDL: low-density lipoproteins; HDL: high-density lipoproteins. Means with different letters in a row differ, *p* < 0.05. N = normal/standard rat chow, PB = plain biscuits, FB = biscuits fortified with PWL and OFSP, PWB = biscuits fortified with PWL only, and SPB = biscuits fortified with OFSP only.

**Table 4:** Effect of treatment on biochemical profile of animals in the control and treated groups.

Parameters	N	PB	FB	PWB	SPB
Urea (mmol/L)	8.73 ± 0.34^b^	6.42 ± 0.60^a^	7.36 ± 0.42^a^	7.62 ± 0.25^a^	6.54 ± 0.47^a^
Creatinine (*µ*mmol/L)	57.50 ± 4.17^a^	56.0 ± 5.78^a^	53.2 ± 2.53^a^	52.40 ± 3.52^a^	61.40 ± 2.75^a^
ALT (U/L)	44.75 ± 2.59^b^	84.0 ± 12.0^a^	62.60 ± 5.61^b^	64.40 ± 7.67^b^	58.60 ± 5.3^a^
Tol protein (mg/dL)	64.25 ± 1.38^b^	56.2 ± 1.46^a^	60 ± 1.14^b^	58.6 ± 0.6^b^	61.2 ± 1.74^a^
Albumin (mg/dL)	25.75 ± 1.49^b^	24.4 ± 1.44^b^	25.8 ± 0.58^b^	25.8 ± 0.49^b^	25.6 ± 0.24^a^
Globulin (mg/dL)	38.5 ± 1.04^b^	30.4 ± 1.63^a^	35.6 ± 1.17^b^	32.8 ± 0.73^b^	35.6 ± 1.96^a^

Data presented are means and standard errors of some biochemical parameters of animal groups. ALT: alanine aminotransferase. Means with different letters differ, *p* < 0.05. N = normal/standard rat chow, PB = plain biscuits, FB = biscuits fortified with PWL and OFSP, PWB = biscuits fortified with PWL only, and SPB = biscuits fortified with OFSP only.

## Data Availability

The data used to support the findings of this study are available from the corresponding author upon request.
